# Nurses’ Experience Regarding Barriers to Providing Internet Plus Continuous Nursing: Mixed Methods Study

**DOI:** 10.2196/65445

**Published:** 2025-07-02

**Authors:** Huanhuan Huang, Zhiyu Chen, Lijuan Chen, Xingyao Du, Qi Huang, Wenbi Jia, Qinghua Zhao

**Affiliations:** 1 Department of Nursing, The First Affiliated Hospital of Chongqing Medical University, 1st Youyi Road, Chongqing, 400016, China, 86 89012206; 2 Nursing Research Centre, The First Affiliated Hospital of Chongqing Medical University, Chongqing, China; 3 Department of Orthopedics, The First Affiliated Hospital of Chongqing Medical University, Chongqing, China; 4 School of Public Health, Chongqing Medical University, Chongqing, China

**Keywords:** transitional care, digital governance, influencing factors, barrier, nursing, mixed methods study, internet, digitalization, China, digital health, self-made questionnaire, interview, Chinese nurses, health care delivery, policymakers, communication, care quality, patient satisfaction

## Abstract

**Background:**

The novel medical model of “Internet Plus continuous nursing” has received much attention under the dual background of aging and digitalization in China. However, there is a scarcity of studies that report on the potential barriers and challenges associated with the implementation of this practice.

**Objective:**

This study aimed to investigate and understand nurses’ experience regarding barriers to providing Internet Plus continuous nursing.

**Methods:**

A sequential mixed methods design was adopted. In the first phase, a self-made questionnaire was used to quantify the barriers and challenges into 3 domains: management, relational, and information continuity. In the second phase, nurses who participated in the Internet Plus continuous nursing program were invited to attend semistructured interviews to further explore, explain, and understand the complexities behind these data, obtaining more detailed information on participants’ experiences, perspectives, and meanings.

**Results:**

A total of 4638 participants from 312 hospitals were selected for the final analysis; the adjusted mean score of the survey was 3.49 (SD 0.83). Among the 3 domains, management continuity had the lowest score (mean 3.32, SD 0.97), followed by relational continuity (mean 3.44, SD 0.9) and information continuity (mean 3.62, SD 0.92). The results of the multivariable analysis showed that age, education level, and a greater number of working years were predictors of continuity for Internet Plus continuous nursing (*P*<.001). Following the qualitive study, 8 subthemes emerged from 72 initial codes and were grouped into 3 themes: organizational changes, practice changes, and future directions.

**Conclusions:**

This mixed methods study revealed that Chinese nurses may have differential challenges when providing Internet Plus continuous nursing, particularly in management continuity. To better benefit patients and improve health care delivery, health care organizations and policymakers should implement strategies to improve interdisciplinary relationships, establish and perfect organizational management, and enhance communication.

## Introduction

Continuous nursing refers to a comprehensive approach to providing uninterrupted care and attention to patients in health care settings [1], combining the continuation of nursing services and time and health services [2]. As a part of overall care and as a core element of high-quality primary care, continuous nursing has been shown to reduce mortality rates and health costs, improve patient satisfaction, and enhance overall health care outcomes [3-5]. However, the traditional continuous nursing model still suffers from some drawbacks and does not comprehensively satisfy the complicated care needs of patients.

With the rapid development of mobile technology and the widespread use of mobile phones and the internet, the traditional continuous nursing model has slowly shifted to what is termed “Internet Plus continuous nursing,” which has gained significant attention. Particularly, the outbreak of the COVID-19 pandemic has reminded the health care system of the importance of creating new strategies, using traditional approaches alongside information technologies [6]. Internet Plus continuous nursing represents a paradigm shift in health care delivery [7], referring to the integration of internet technology with continuous nursing care or monitoring [8]. This approach aims to build a connection between health care providers and patients by leveraging the power of the internet to facilitate real-time monitoring, communication, and data sharing. Health care professionals can thus remotely monitor patients’ vital signs, provide guidance, offer medication reminders, and deliver personalized care plans [9,10]. In addition, the novel model could also streamline clinical workflows [11], enhance patient engagement [12], and facilitate knowledge sharing [13]. The Chinese government and industry attach great importance to the development of this work and have introduced multiple policies to provide further guidance, such as the National Nursing Development Plan (2021‐2025) [14] and the Action Plan for Further Improvement of Care Services (2023‐2025) [15]. Driven by strong policy support, the initiative has garnered widespread attention from health care institutions and the public.

However, Internet Plus continuous nursing is still in the pilot stage in China and primarily targets key populations such as discharged patients, maternal and infant groups, and patients with chronic diseases [15]. For example, data from one Chinese province revealed that 85.45% of hospitals have yet to adopt “Internet Plus nursing services,” despite 97.87% of nurses expressing a willingness to participate [16]. Only 14.55% of nurses are currently engaged in such services within their hospitals. Even among hospitals that have implemented these programs, gaps persist between service offerings (eg, scope, scheduling, continuity) and actual market demand [17].

Although prior research has examined the factors influencing nursing care continuity [18,19], few have specifically examined Internet Plus continuous nursing. Existing research has investigated nurse willingness and program effectiveness [20,21], yet there is a critical gap in understanding the implementation barriers and challenges, particularly in primary hospitals, where logistical and resource constraints may amplify these issues. Identifying these barriers from the perspective of participating nurses is essential not only for preparedness but also for improving service quality.

Therefore, the aim of this mixed methods study is to investigate the factors influencing the implementation of Internet Plus continuous nursing across primary, secondary, and tertiary hospitals, with a specific focus on barriers. The findings of this study will provide valuable insights for policymakers, health care administrators, and nursing practitioners, enabling them to develop strategies and interventions that can strengthen the continuity of care in the digital era.

## Methods

### Ethical Considerations

Ethics approval was obtained from the First Affiliated Hospital of Chongqing Medical University’s research ethics committee (2023-34). All procedures complied with the Declaration of Helsinki.

Written informed consent was obtained from all participants prior to their involvement in the study. For online interviewees, verbal consent was obtained and documented. Participants were explicitly informed of their right to withdraw from the study at any time without penalty. No secondary data analysis was conducted in this study.

All collected data were deidentified prior to analysis. Personal identifiers were removed and replaced with coded numbers to protect participant confidentiality. Electronic data were stored on password-protected servers with access restricted to the research team.

Participants did not receive financial compensation for their involvement in this study. However, they were provided with the study results upon request and had access to any potential benefits emerging from the research findings.

### Quantitative Study

#### Participants

Participants were convenience sampled from January 2021 to March 2021 in Chongqing, China. The inclusion criteria were: (1) nurses directly involved in Internet Plus continuous nursing services, (2) who had over 1 year of experience and (3) provided informed consent and were willing to participate. The exclusion criteria were: (1) visiting nurses who were not affiliated to the hospital and (2) nurses who were on vacation during data collection.

#### Measurement Tools

A 14-item questionnaire specifically tailored to health care professionals was developed based on the Continuity of Care model [22], with reference to patient-reported measures [23,24]. The questionnaire was constructed using the Delphi method (Multimedia Appendix 1), demonstrating strong expert consensus with authoritative coefficients (Cr) of 0.85 and 0.91 for the 2 rounds, respectively. The instrument comprises 3 domains, namely relational (5 items; Cronbach coefficient=0.832), information (3 items; Cronbach coefficient=0.865), and management (6 items; Cronbach coefficient=0.840) continuity, with a good content validity index (0.96) and an overall Cronbach coefficient of 0.871. Each item was scored from 1 (“Rarely”) to 5 (“Always”) on a 5-point Likert scale; higher scores reflect higher quality and efficiency of implementation of Internet Plus continuous nursing.

#### Data Collection

The questionnaire was distributed to hospitals through an online survey tool, Wenjuanxing [25], by the Chongqing Hospital Association. The hospital manager then sent the link to Internet Plus continuous nursing–related health care providers. On the first page of the questionnaire, the objective, process, and anonymization of data were introduced in detail. Individuals who received the link were also requested to provide informed consent. In addition, only those who clicked “accept” had access to the full questionnaire.

#### Statistical Analysis

The software SPSS version 26.0 (IBM Corporation) was used for analysis, the measurement of normal distribution data were described by means and SDs, and 2-tailed independent sample *t* tests were used for comparison between groups. The count data were represented by the use case (%), and a chi-square test was used for the comparison between groups. The test level was bilateral (α=.05).

### Qualitative Study

#### Participants

Participants (referred to as as P1, P2, and so on, up to P15) were selected using purposive and snowball sampling methods. The inclusion and exclusion criteria were the same as those for the quantitative study. To meet the maximum variation, the level, setting, and Internet Plus continuous nursing experience of the hospital were taken into account. Recruitment ceased once theoretical saturation was reached (ie, when no new codes and themes emerged from the data) [26]. Finally, a total of 15 participants were recruited (Table 1).

**Table 1.** Demographic information of participants in the qualitative study.

	Level of hospital	Age (years), n	Professional title	Working years, n
P1	Tertiary	49	Vice-senior	27
P2	Tertiary	32	Senior	31
P3	Tertiary	46	Vice-senior	27
P4	Secondary	48	Vice-senior	30
P5	Secondary	55	Vice-senior	32
P6	Secondary	47	Vice-senior	29
P7	Secondary	52	Middle	29
P8	Secondary	49	Middle	27
P9	Secondary	48	Middle	26
P10	Primary	47	Vice-senior	27
P11	Primary	50	Middle	29
P12	Primary	53	Middle	31
P13	Primary	48	Primary	28
P14	Primary	56	Vice-senior	36
P15	Primary	52	Middle	32

#### Interview Outline

The intention of holding interviews was to further explore, explain, and understand the complexities behind these data, obtaining more detailed information on participants’ experiences, perspectives, and meanings. An interview outline containing 4 questions was used to structure the interview (Table 2). The outline was formed through literature review [27,28] and the quantitative study results, and pilot-tested with 4 participants to ensure the quality and feasibility.

**Table 2.** Interview outline.

Interview guideline	Detailed inquiry
Please provide a brief introduction to the current development of Internet Plus continuous nursing services at your esteemed institution.	What was the driving force behind the initiation of this endeavor? Why was it not implemented? What challenges were faced?
As an implementer, what obstacles do you think have been encountered during the process?	Could you share your views on “information continuity”? Could you share your views on “organizational continuity”? Could you share your views on “management continuity”?
As an implementer, what factors do you think have facilitated the process?	In your opinion, how should these facilitating factors be implemented?
What advice would you give to organizations that have not yet implemented Internet Plus continuous nursing services?	—^a^

a Not applicable.

#### Data Collection

Descriptive qualitative research methods were applied and data were collected through semistructured interviews. Each interview was conducted by the first and second authors, who have both received qualitative research training and have experience interviewing. Due to the impact of the pandemic, interviews were conducted using a combination of online (n=5) and offline (n=10) approaches. Prior to the interviews, the purpose, content, and methods of the study were communicated to the participants. For all interviewees, informed consent was obtained from before the interviews commenced. A quiet and independent space was chosen and sufficient time was provided for the interviews. Similarly, online interviews were scheduled based on a mutually agreed upon time, and participants were advised to select a quiet environment in an independent space and were provided with ample time. The online interviews were conducted via WeChat video calls, and video recordings were taken after obtaining informed consent from the interviewees. Throughout the interviews, a neutral stance was maintained by the interviewers, encouraging the participants to express themselves fully. When necessary, their viewpoints were clarified and confirmed. Note-taking and audio recording were performed during all interviews.

#### Statistical Analysis

A qualitative content analysis was used [29], comprising several rigorous steps:

1. Data processing: 2 researchers independently transcribed the raw data and generated initial codes. A third researcher verified the coding consistency and accuracy. The team discussed discrepancies until a full consensus was reached.
2. Thematic development: a researcher conducted higher-level analysis by synthesizing codes into meaningful categories and identifying emerging themes through iterative comparison [30]. Data collection continued until theoretical saturation was achieved (ie, no new codes emerged after 3 consecutive interviews and established themes demonstrated sufficient depth and variation) [26].
3. Validation procedures: all research team members participated in regular peer debriefings to compare interpretations of themes and subthemes and resolve any analytical disagreements through discussion. Member checking was implemented by sharing interview summaries with participants and incorporating their feedback to ensure the accurate representation of experiences [31].

## Results

### Quantitative Study

Overall, a total of 4911 health care providers from 312 institutions received the questionnaire, while only 4638 were selected for the final analysis (response rate=94.44%); 98 were excluded according to the inclusion and exclusion criteria, 135 were excluded for incomplete answers, and, to control quality, 40 were excluded for response times outside the 5th-95th percentile range.

As shown in Table 3, respondents were predominantly from tertiary hospitals (n=2630, 56.71%) or secondary hospitals (n=1883, 40.60%), and most were female (n=4505, 97.13%). The largest age group was 26‐35 years (n=2750, 59.29%). Notably, older participants (≥46 years) accounted for 6.81% (n=316) but had the highest continuity scores (mean 91.0, SD 10.9). Most held an undergraduate degree (n=3386, 73.01%), while only 0.52% (n=24) had received graduate-level education. Job titles were primarily primary-level (n=2768, 59.68%), and the majority had 5‐9 years of work experience (n=2679, 57.76%). Those with ≥10 years of experience (n=1039, 22.40%) reported significantly higher continuity scores (mean 84.1, SD 11.5).

**Table 3.** Demographic characteristics and multivariable analysis.

Variables	Participants, n (%)	Continuity score, mean (SD)	Coefficient (multivariable)	
			β (95% CI)	*P* value
Hospital level				
Primary	125 (2.70)	74.8 (10.7)	Reference	—^a^
Secondary	1883 (40.60)	76.1 (11.2)	0.76 (–1.21 to 2.73)	.45
Tertiary	2630 (56.71)	77.6 (12.3)	1.73 (–0.22 to 3.69)	.08
Sex				
Male	133 (2.87)	76.5 (11.9)	Reference	—
Female	4505 (97.13)	76.9 (11.9)	–1.70 (–3.59 to 0.19)	.08
Age (years)				
<25	670 (14.45)	91.0 (10.9)	Reference	—
26‐35	2750 (59.29)	72.5 (10.6)	16.39 (14.05 to 18.74)	<.001
36‐45	902 (19.45)	75.0 (11.1)	–14.81 (–16.78 to –12.84)	<.001
≥46	316 (6.81)	81.4 (10.5)	–9.47 (–11.09 to –7.85)	<.001
Education level				
College and below	1228 (26.48)	76.3 (12.5)	Reference	—
Undergraduate	3386 (73.01)	77.2 (11.6)	–1.12 (–1.96 to –0.29)	.008
Graduate	24 (0.52)	72.8 (11.4)	–6.38 (–10.81 to –1.94)	.005
Job title				
Primary	2768 (59.68)	74.8 (11.4)	Reference	—
Middle	1497 (32.28)	78.6 (11.5)	–0.36 (–1.20 to 0.47)	.40
Advanced	373 (8.04)	86.0 (11.6)	–1.64 (–3.32 to 0.05)	.05
Working years				
≤2	365 (7.87)	72.6 (10.7)	Reference	—
2~4	555 (11.97)	72.8 (11.3)	–0.15 (–1.65 to 1.35)	.85
5~9	2679 (57.76)	75.6 (11.1)	1.94 (0.30 to 3.59)	.02
≥10	1039 (22.40)	84.1 (11.5)	3.94 (1.88 to 6.00)	<.001

a Not applicable.

Among the continuity domains, the information domain scored highest (mean 3.62, SD 0.92), followed by the relational domain (mean 3.44, SD 0.90) and management domain (mean 3.32, SD 0.97). The overall mean continuity score was 3.49 (SD 0.83), indicating moderate-to-high levels of perceived continuity. The results of multivariable analysis showed that age, education level, and a greater number of working years were predictors of continuity for Internet Plus continuous nursing (*P*<.05).

### Qualitative Study

**Figure 1.**
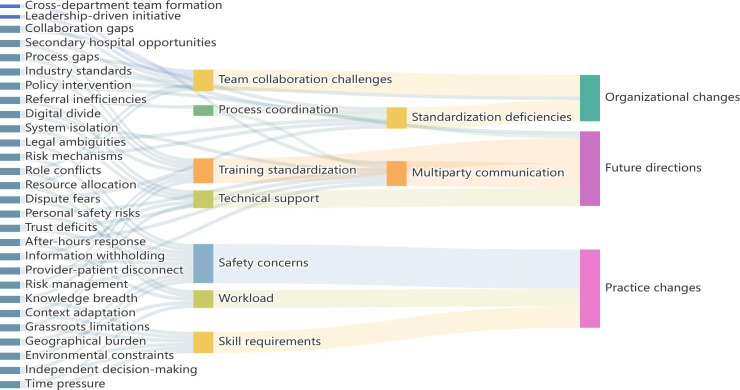
Three-level coding framework for Internet Plus continuity care. Data were derived from the qualitative analysis of 15 health care providers.

The initial open coding identified 72 initial codes (eg, “Process gaps,” “Legal ambiguities”), which were then clustered into 8 subthemes (eg, “Team collaboration challenges,” “Safety concerns”). After verification by team members, these were consolidated into 3 final themes, as illustrated in Figure 1.

#### Theme 1: Organizational Changes

##### Team Collaboration Challenges

Participants reported significant difficulties in establishing and sustaining interdisciplinary teams for Internet Plus continuity care, citing role ambiguity, resource disparities, and institutional resistance. P11 described the lack of structured team development: “We assembled staff from different departments overnight without clear role assignments. This meant starting everything from scratch.” The absence of transitional frameworks exacerbated role confusion, particularly in lower-resource settings. P13 emphasized disparities in institutional capacity: “How can we match tertiary hospitals? Our IT team consists of only 1.5 full-time staff maintaining basic systems.” Functional teamwork remained elusive even when teams were formally established. P4 noted systemic neglect: “The IT department never attends our meetings. When systems fail, we are told, ‘This is not a priority.’”

##### Standardization Deficiencies

Participants across all hospital tiers identified critical gaps in clinical protocols, legal safeguards, and financial sustainability, which collectively hindered Internet Plus continuity care implementation. Tertiary hospitals developed internal protocols, but these were rarely adopted by primary or secondary institutions. P3 stated, “We created our own post-discharge monitoring checklist, but it is ineffective if community clinics do not use it.” P2 raised critical questions about accountability: “If a patient deteriorates after a virtual consultation, who is responsible—the platform, the nurse, or the hospital? Without clear liability frameworks, many nurses as well as patients hesitate to engage fully.” Variability in care quality was another challenge, particularly in resource-limited settings. P12 noted, “Our rural patients expect hospital-level care at home, but with no training protocols, outcomes vary wildly.” Financial sustainability emerged as a major barrier too. Over 80% (12/15) of participants reported providing Internet Plus continuity care as an uncompensated service due to unclear reimbursement policies. P15 contrasted this with privatized models: “Affluent urban hospitals charge fees through third-party apps, but our rural patients cannot afford to pay. Shouldn’t the government subsidize this essential service?”

### Theme 2: Practice Changes

#### Workload

The expansion of Internet Plus continuous nursing extends the reach of nursing services, enhancing patient care accessibility. However, in settings with existing nursing shortages, clinical staff face increased workloads due to additional responsibilities, training demands, and after-hours patient interactions. Nurses must balance traditional duties with Internet Plus continuous nursing tasks, leading to role overload. Most institutions lack specialized roles for this project, forcing existing nurses to absorb extra tasks. P1 stated, “After joining the team, I not only need to deal with the previous affairs, but also need to spend extra personal time to study and attend training, which increases the workload virtually.” Patients often seek support outside standard working hours, contributing to burnout. “The frequency of overtime work is higher, because some patients may contact you after work hours” (P15). Home visits for remote patients also add physical and logistical strain. “Some patients live in a faraway address, travel a long way, one after another cause people are also very tired” (P4).

#### Safety Concerns

In interviews, participants reported a wide range of worries and insecurities. First and foremost was the safety of medical staff. P7 stated, “At the beginning, I was very worried about going to a strange family to provide services, afraid of accidents,” and P9 added, “I prefer to interact with discharged patients who I manage, and we know each other better. For strange orders, I will have some concerns, so before the service is carried out, I will generally communicate well in advance.” Secondly, some respondents reported their own practice safety: “Some family environments have restrictions, lack of necessary space, equipment or drugs, and sometimes I even have no suitable place to carry out operations” (P10) and “I am very worried that some patients and their families do not take the initiative to report the history of allergy and infection because of their own reasons, which is very dangerous for patients and myself” (P14). Finally, some respondents cited concerns about medical disputes: “Although it hasn’t happened yet, I hope it never will... What should I do in the event of a patient’s death or medical malpractice?” (P6).

#### Skill Requirements

Implementing Internet Plus continuity services requires a higher level of competence from health care professionals, but in practice, most providers still report having their own challenges. Nurses often face questions or requests outside their specialization, requiring ad hoc problem-solving without immediate support. “This is not an individual case, when you provide a specialized service, the patient or family may ‘politely’ ask you some other questions, which may be beyond your expertise,” stated P3. Unlike hospital settings, Internet Plus continuous nursing sometimes requires solo decision-making with a few colleagues or specialists for consultation. “This is very different from the hospital scene, you know that your teammates are around you, but at this time, you may be alone” (P11). Older or rural patients struggle with technology, forcing nurses to double as tech support. P13 noted, “Half my visit time is spent teaching older patients how to use the app. They forget by the next week, and we start over.”

### Theme 3: Future Directions

#### Multiparty Communication

Participants observed in their clinical practice that Internet Plus continuous nursing involves multiple stakeholders, and that there is a need to strengthen communication to ensure the smooth implementation of services. Patients often distrust community hospitals’ capabilities, viewing them as limited to basic services (eg, health education), while overestimating tertiary hospitals’ roles in continuous care. P3 suggested that “If community clinics adopt our monitoring tools, patients would see them as equal partners in care.” Nurses reported inefficiencies when transferring patients between tertiary, secondary, and primary care due to unfamiliarity with the community teams. P8 stated, “Let patients meet ‘their’ community nurse before discharge—it humanizes the transition.” Additionally, participants also admitted that while the model expands care accessibility, it also depersonalizes care, eroding rapport and leading to miscommunication. P5 provided the following insight: “We need to learn how to make ‘digital warmth’—emojis aren’t enough.”

#### Technical Support

Although the current level of informatization varies significantly across health care institutions in China, interviewees expressed optimism about the potential for technical support to enhance the implementation of Internet Plus continuous care in the future. Participants emphasized that addressing existing disparities and investing in robust digital infrastructure could pave the way for the seamless integration of services. Several participants highlighted the need for a centralized patient record system as a cornerstone of future technical advancements. P13 suggested that “A centralized patient record system would eliminate duplicate tests and lost information.” This sentiment was echoed by P4, who added, “In the future, we should aim for region-level interoperability so that patient data flows seamlessly across different levels of care.” Additionally, participants expressed excitement about the potential of emerging technologies, such as artificial intelligence, big data analytics, and wearable devices, to transform continuous care. P7 offered a forward-looking view: “These innovations will require substantial investment and collaboration, but they hold immense promise for improving outcomes and reducing costs.”

#### Training Standardization

Skill gaps, particularly in primary and secondary care settings, were a major concern. P3 noted, “Personnel are unprepared for new challenges, including risk prediction and process adaptation.” P7 suggested targeted training in areas like data literacy and digital tools to bridge these gaps. Managing risks such as data breaches and miscommunication was seen as critical. P14 recommended training in risk prediction and mitigation. P9 added, “Predictive analytics tools can help nurses identify high-risk patients and intervene proactively.” To ensure practical application, experiential learning and peer networks were suggested. P4 advocated for simulations, while P15 highlighted the value of collaborative platforms for sharing best practices.

## Discussion

### Principal Findings

In the context of aging populations and rapid digitalization, Internet Plus continuity care has garnered significant attention in China from both scholars and clinicians. To our knowledge, this is the first mixed methods study exploring nurses’ experiences with Internet Plus continuity care services in China. Our research not only identified the most challenging aspects of implementation through objective measurements but also delved into the underlying reasons via semistructured interviews.

Consistent with prior studies [32], our results show that information continuity scored highest among the 3 dimensions of the Continuity of Care model. This aligns with the consensus that seamless information flow is critical for care continuity, with technology platforms serving as its backbone [33]. However, our qualitative data reveal persistent gaps: participants highlighted poor interoperability between institutional systems and emphasized the need for remote assistance. These findings are further supported by evaluations of 17 Internet Plus nursing service applications in China, where most platforms were rated “average” or “poor” in credibility (82%) and demand fulfillment (94%) [34]. To address these issues, future platform development should adopt a co-design approach, ensuring that the needs of both health care providers and recipients are prioritized [35].

Furthermore, our research demonstrates that relational continuity scores exhibit average performance. While continuity of care relies on stable, trusting relationships between patients and providers [36], it also depends on effective interdisciplinary collaboration [37]. Nurses play a pivotal role in sustaining continuity, even in physician-driven services [38], yet hierarchical nursing teams often struggle with complex supervision and authorization processes. This leads to diminished confidence among nurses [39,40], as reflected in our interviews. To mitigate this, preservice training, ongoing education, and structured support are essential to empower nurses in multidisciplinary teamwork and task delegation.

The most significant barrier identified was management continuity, mirroring the findings of Roy et al [41], who cited administrative burdens as a primary obstacle. In the Internet Plus context, these challenges appear amplified. Nurses in our study prioritized safety, income security, and collaboration with patients and their family—concerns echoed in prior research [8,42]. Safety risks, in particular, stem from perceived inadequacies in personal competency, lack of prescription authority, insufficient emergency equipment, and limited team support [43,44]. Notably, while shared nurses demonstrated moderate emergency response capabilities, their recovery capacity scored lowest [45]. Cross-sectional studies suggest that successful implementation hinges on insurance coverage, hospital-affiliated licensing, clear contractual terms, real-time location tracking, and emergency alert systems [46].

Our work clearly has some limitations. First, the questionnaire was self-developed and lacks external validation, though qualitative insights help counterbalance this concern. Future research should employ validated tools, such as the scale by Tian et al [47], for comparative analysis. Second, while all interviewees had experience with Internet Plus continuity care, the perspectives of nonparticipating nurses remain unexplored and warrant investigation. Despite these limitations, our findings provide actionable insights for improving the quality and scalability of Internet Plus continuous nursing services in China.

### Conclusion

This study addresses a significant gap in the literature by investigating the barriers and challenges faced by nurses in providing Internet Plus continuous nursing, a novel medical model that has gained considerable attention under the dual background of aging and digitalization in China. Our findings revealed that Chinese nurses may have differential challenges when providing Internet Plus continuous nursing, particularly in management continuity. We recommend that, to better benefit patients and improve health care delivery, health care organizations and policymakers implement strategies to improve interdisciplinary relationships, establish and perfect organizational management, and enhance communication.

## Supplementary material

10.2196/65445 Multimedia Appendix 1 Basic information of experts collected via the Delphi survey.
