# Supplementary material for: Nurses’ Experience Regarding Barriers to Providing Internet Plus Continuous Nursing: Mixed Methods Study
Source: JMIR Med Inform. 2025 Jul 2;13:e65445. doi: 10.2196/65445 (PMC12239685; doi:10.2196/65445)
Supplement: Multimedia Appendix 1 [file medinform-v13-e65445-s001.docx]

### Appendix 1. Basic information of experts involving in Delphi survey

| **No.** | **Age (years)** | **Job** | **Education level** | **Research interest** | **Working years** |
| --- | --- | --- | --- | --- | --- |
| 1 | 53 | Head nurse | Undergraduate | nursing management | 34 |
| 2 | 38 | Clinical nurse | graduate | Surgical nursing | 10 |
| 3 | 33 | Clinical nurse | graduate | statistics | 6 |
| 4 | 52 | Head nurse | Undergraduate | Community nursing | 32 |
| 5 | 56 | Head nurse | Undergraduate | Obstetrics nursing | 37 |
| 6 | 48 | Head nurse | graduate | Geriatric nursing | 24 |
| 7 | 49 | Clinical nurse | graduate | Patient safety | 25 |
| 8 | 43 | Head nurse | graduate | Critical nursing | 20 |
